# Pitch enhancement facilitates word learning across visual contexts

**DOI:** 10.3389/fpsyg.2014.01468

**Published:** 2014-12-22

**Authors:** Piera Filippi, Bruno Gingras, W. Tecumseh Fitch

**Affiliations:** Department of Cognitive Biology, Faculty of Life Sciences, University of ViennaVienna, Austria

**Keywords:** cross-situational learning, prosody, word learning, sound-meaning mapping, infant directed speech, language evolution

## Abstract

This study investigates word-learning using a new experimental paradigm that integrates three processes: (a) extracting a word out of a continuous sound sequence, (b) inferring its referential meanings in context, (c) mapping the segmented word onto its broader intended referent, such as other objects of the same semantic category, and to novel utterances. Previous work has examined the role of statistical learning and/or of prosody in each of these processes separately. Here, we combine these strands of investigation into a single experimental approach, in which participants viewed a photograph belonging to one of three semantic categories while hearing a complex, five-word utterance containing a target word. Six between-subjects conditions were tested with 20 adult participants each. In condition 1, the only cue to word-meaning mapping was the co-occurrence of word and referents. This statistical cue was present in all conditions. In condition 2, the target word was sounded at a higher pitch. In condition 3, random words were sounded at a higher pitch, creating an inconsistent cue. In condition 4, the duration of the target word was lengthened. In conditions 5 and 6, an extraneous acoustic cue and a visual cue were associated with the target word, respectively. Performance in this word-learning task was significantly higher than that observed with simple co-occurrence only when pitch prominence consistently marked the target word. We discuss implications for the pragmatic value of pitch marking as well as the relevance of our findings to language acquisition and language evolution.

## INTRODUCTION

A crucial issue in the study of word learning is the inherent uncertainty of the referential act of naming in sound-meaning associations (Quine, 1960), sometimes called the “Gavagai!” problem. Both the child acquiring spoken language and the adult learning a new language have to map sounds onto referents, a problem that involves the triple challenge of (a) extracting (i.e., identifying and remembering) a word out of a continuous sound sequence, (b) inferring one or more possible referents within the current visual scene, and (c) mapping the segmented word onto its broader intended referential/pragmatic meaning(s), and/or grammatical role(s) (Bloom, 2000). The final step includes the possibility of extending the reference over a potentially infinite set of instances of the same semantic category (Brown, 1958; Waxman and Gelman, 2009), and to an open-ended set of novel utterances (Chomsky, 2000).

Language learners might infer the referential meaning of the spoken words by hearing them in various contexts of use (Wittgenstein, 2009), and by using multiple pragmatic or linguistic cues such as eye gaze (Nurmsoo and Bloom, 2008), discourse novelty, syntax (Wagner and Watson, 2010), and tactile interaction (Seidl et al., 2014). Here we focus on two important sources of information for word learning: cross-situational statistics and prosodic cues in the speech signal. Most research has investigated the role of these two cues separately (Morgan et al., 1987; Medina et al., 2011; Shukla et al., 2011; Vlach and Sandhofer, 2014). In the present study we simulate the complexity of real-world word learning processes in the laboratory, and bring research on prosody and statistical learning together. Specifically, our study builds on three key findings from previous research: (i) cross-situational statistical regularities, expressed as co-occurrence between labels and their intended referent across different visual scenes, favor learning of conventionally defined sound-meaning associations (Yu and Smith, 2007), (ii) the statistically regular co-occurrence between a target word and its intended referent through learning trials facilitates object categorization, i.e., the extension of target words to multiple exemplars of the visual referent (Waxman and Braun, 2005); and (iii) the exaggerated pitch parameter cross-culturally employed in infant-directed speech (IDS) provides markers of acoustic salience that guide selective attention and are often used to highlight target words (Grieser and Kuhl, 1988; Fernald and Mazzie, 1991; Aslin et al., 1996).

Based on these findings, we test the prediction that marking target words with IDS-typical pitch differential contrasts plays a key role in supporting word learning across different visual scenes. Although numerous studies have addressed the positive effect of speech directed to infants or strangers in conveying language-specific phonological information (Burnham et al., 2002; Kuhl, 2004), as cues to word segmentation (Thiessen et al., 2005; Shukla et al., 2011), or as cues to the syntactic structure of the sentence (Sherrod et al., 1977), no research we know of has investigated the effects of IDS-typical emphatic stress of single target units in the service of word learning, thus extending beyond the first step of sound extraction or single object labeling. The present study aims at filling this gap. With this overall aim, the research reported here specifically compares the learning effects of IDS typical pitch emphasis with those of other visual and acoustic attentional cues.

### A NEW PARADIGM FOR THE INVESTIGATION OF WORD LEARNING: THE EIM TASK

Much previous research in word learning has addressed the acquisition of sounds spoken in isolation in association with objects represented in pictures isolated from any surrounding visual scene (Gleitman, 1990; Markman, 1990; Baldwin, 1993). But such paradigms greatly simplify what actually happens in natural learning situations, which typically include an indefinite number of potential referents (Medina et al., 2011), and where the target words are typically spoken not in isolation, but in connected discourse, within a sequence of continuous sounds. Thus, in a real label-referent mapping situation, learners have to somehow identify the key word(s) to be linked to the visual scene.

To address these issues, we introduce a new paradigm for studying word learning, which we call the Extraction/Inference/Mapping (EIM) task (target sound string Extraction, referential category Inference, and label-meaning Mapping). This paradigm uses photographs of complex visual scenes, providing a naturalistic visual parsing challenge that poses Quine’s problem of indeterminacy of the intended referent (Quine, 1960) in a laboratory environment. Simultaneously, a stream of spoken words is presented acoustically.

**FIGURE 1:**
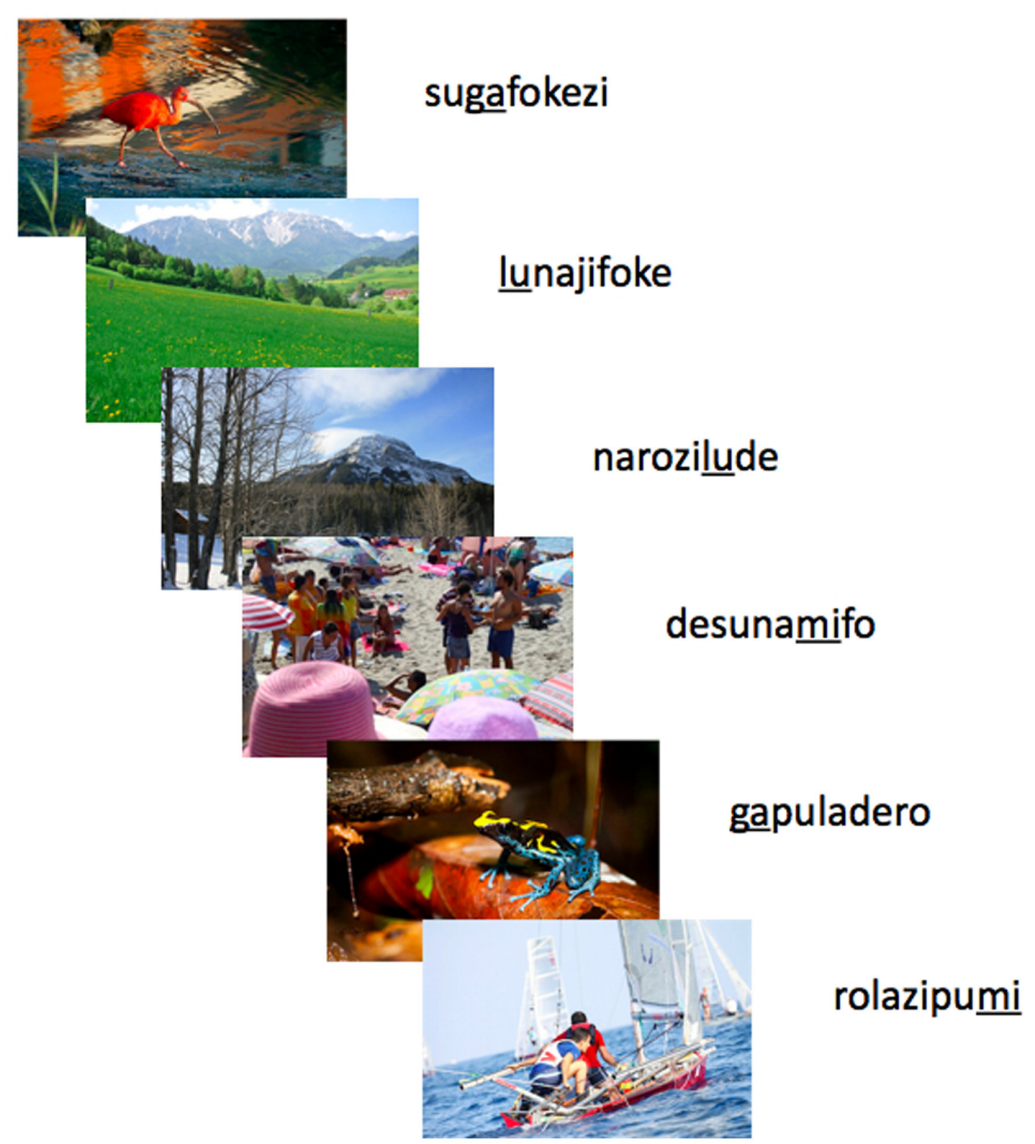
**Example stimuli presentation series.** In each experimental condition, participants were exposed to 45 successive stimuli, consisting of images paired with an auditory utterance of five monosyllabic words. Each image category – humans, non-human animals, mountains – was linked only to a specific word (“target label”) randomly assigned to that referential category (different for different subjects). In this example, /mi/ always co-occurs with the category “humans,” /ga/ with “non-human animals,” and /lu/ with “mountains” (underlined in the figure). Due to copyright and legal concerns, in this example we used copyright-free pictures instead of the original pictures from the National Geographic Website.

To control the key features of the auditory stimuli to which learners are exposed, we created an artificial language made of non-sense monosyllabic words (cf. Gomez and Gerken, 2000). Each utterance in this artificial language is a stream of five monosyllabic words containing a single target word (“target label” hereafter) at an arbitrary position. These target labels are consistently associated with the intended category of the photograph (**Figure 1**). Participants must identify the target labels within the speech stream, infer the intended referent category from the photographs, and link these two together into a label-meaning pair which allows them to subsequently extend the acquired word to novel utterance contexts and to new instances of the intended referential category (novel images).

The EIM task can be used with children or adults (and potentially animals). It uses computer-modified natural speech to provide precise acoustic control, and allows for both explicit and implicit learning approaches. The paradigm can be varied in many ways to address multiple questions concerning word learning and language acquisition.

## MATERIALS AND METHODS

### ETHICS STATEMENT

The experiment reported in this article was conducted in accordance with Austrian law and the policies of the University of Vienna. According to the Austrian Universities Act 2002, the appointment of ethics committees is required only for medical universities engaged in clinical tests, the application of new medical methods, and/or applied medical research on human subjects. Accordingly, ethical approval was not required for the present study. Nevertheless, all participants gave written informed consent and were aware that they could withdraw from the experiment at any time without further consequences. All data was stored anonymously.

### EXPERIMENTAL CONDITIONS: OVERVIEW

**FIGURE 2:**
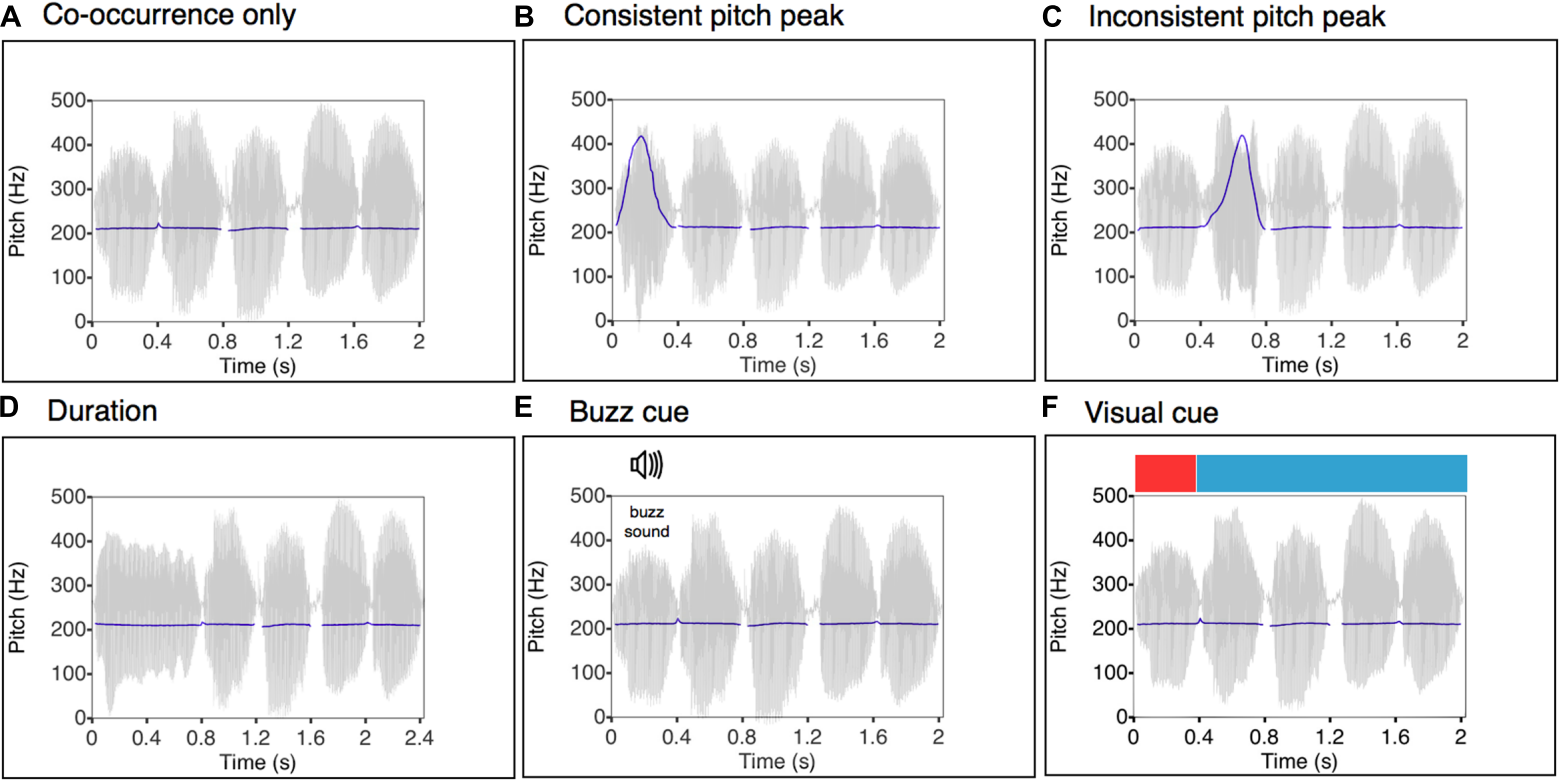
**Speech waveforms and pitch contours corresponding to the utterance “minajifoke” (where the target label was “mi”) as synthesized in each experimental condition. (A)** *Co-occurrence only*: here the pitch contour is flat; the only cue to word learning is the consistent co-occurrence between target labels and their respective visual referents. **(B)** *Consistent pitch peak*: a consistent pitch emphasis marks each target label. **(C)** *Inconsistent pitch peak*: pitch emphasis marks a random word of the utterance. **(D)** *Duration*: a temporal length increase marks each target label. **(E)** *Buzz cue*: the attentional cue during the target label is a buzz sound played from the left channel of the head phones, precisely in correspondence, and for the duration of, the target labels. **(F)** *Visual cue*: the target labels are highlighted by an abrupt temporary color change in the background screen, which is synchronized with the duration of each target label.

Six different experimental conditions were tested (see **Figure 2**). In each condition, the set of referential images was the same, but the signal was manipulated in different ways to provide cues to the location of the target label in the speech stream:

(1) *Label/Category cross-situational co-occurrence only (hereafter Co-occurrence only).* The only cue that can support successful learning in this minimal version of the EIM task is the statistical regularity created by the consistent co-occurrence of the target label with the corresponding image category. This level of information is present in all subsequent experimental conditions, and we refer to it as the “statistical cue.”
(2) *Co-occurrence + consistent pitch peak on target label (hereafter Consistent pitch peak).* A pitch differential was used as a consistent *perceptual-attentional spotlight* to the target label in addition to the statistical cue. We used a large pitch deviation of one octave, a magnitude cross-linguistically typical of IDS (Fernald, 1992). The manipulation of pitch cues typically employed in IDS allows us to examine the specific role of pitch in the process of word learning, which we hypothesized would enhance the effect of the pure cross-modal co-occurrence cue.
(3) *Cross-situational co-occurrence + pitch peak on random word (hereafter Inconsistent pitch peak).* While the target label still co-occurred with its associated image category, the pitch peak in this condition was placed on a random word in each utterance. Consequently, the two cues, namely pitch stress and cross-modal co-occurrence, are inconsistent. By highlighting non-target words, we can explicitly evaluate the hypothesis that IDS-typical pitch excursions simply increase arousal and enhance attention to the speech, irrespective of any specific words’ role or meaning (Fernald, 1992). This condition provides the experimental analog of a learning context in which the speaker wants to teach, say the word “dog,” by using it in different sentences (each in co-occurrence with an instance of the referent “dog”). Given for instance the sentences “This dog is brown,” “That dog over there is mine,” “I think this dog is cute,” this experiment examines what would happen when highlighting a non-target word within each utterance (e.g., “brown,” “mine,” “think”), but also occasionally highlighting “dog.”
(4) *Cross-situational co-occurrence + increased duration of the target label (hereafter Duration).* A prominent duration contrast – doubling the target label’s length – was employed to provide a non-pitch vocal cue to the target label.
(5) *Cross-situational co-occurrence + extraneous acoustic cue (hereafter Buzz cue).* A low-frequency, extraneous acoustic cue (an 80 Hz buzz) was played simultaneous with the target label, providing a non-speech acoustic cue to the target label.
(6) *Cross-situational co-occurrence + visual cue (hereafter Visual cue).* A visual cue – a prominent change in the color of the screen behind the presented image, from blue to red – provided a consistent visual cue to the identity of the target label.

Our manipulation of these different types of sensory information as selective attention markers to the target label allowed us to examine whether pitch enhancement has a special status in facilitating word learning.

### PARTICIPANTS

For each condition, 20 individuals at the University of Vienna were recruited via posters or Internet advertisement, for a total of 120 adult participants (71 females and 49 males, mean age = 23.7, range = 18–37) in a between-subjects design. Custom software (Experimenter version 3.5) written in Python 2.6 was used to present the stimuli and collect mouse-click responses. Participants were given modest monetary compensation or candy in exchange for their participation in this short (roughly 8 min) experiment.

### MATERIAL

The stimuli consisted of photographed images, presented on an LCD monitor, paired with artificial language utterances presented over headphones.

(1) Images. Forty-five unique full-color images of real life scenes were selected, each depicting one of three intended semantic categories: humans, mountains and non-human animals (“animals” hereafter). The images were downloaded from the National Geographic website (http://www.nationalgeographic.com), and scaled to 300 × 300 pixels. Care was taken that no obvious emotional or written content was depicted in these pictures.
(2) Sounds. Strings of five CV (consonant + vowel) words (our artificial language “utterances”) containing the target label at a random position in the string were presented. 45 utterances were subdivided into three different sets of 15, each of which referred to one of the three image categories (see supplemental data online). Each set shared one distinctive word that consistently occurred in association with the corresponding image category. The target labels were /mi/, /ga/, and /lu/ and the image set to which they were paired was varied randomly for each participant. The position of the target label was varied systematically across each utterance, appearing in each of the five “slots” with equal frequency (Kuhl, 2004). Otherwise, all other words of the utterances were treated as “stems” that were systematically shared across the three utterance sets, and which therefore had no consistent referential link to the visual stimuli. Hence, only the words shared within each utterance set constituted statistically valid target labels.

In order to avoid co-articulation between adjacent words, as in Johnson and Jusczyk (2001), each word was recorded individually. Each word was then acoustically modified in PRAAT (Boersma and Weenink, 2007). In particular, the words’ pitch and duration were modulated using the pitch synchronous overLap-add (PSOLA) algorithm. Word amplitude was made consistent: each word’s intensity was adjusted to mean 70.0 dB (SD = 0.2) relative to peak amplitude. Except for the “duration” condition, the duration of each word was normalized (mean 400 ms; SD = 2 ms).

#### Perceptual manipulation of the signal in each experimental condition

***Co-occurrence only.*** The pitch-, loudness- and duration-norma lized words were concatenated without pauses to form five-word utterances. In this first condition the target labels’ pitch was normalized to have the same F0 as the four other words (*M* = 210.7 Hz; SD = 0.6 Hz).

***Consistent pitch peak.*** The target label was manipulated to have a much higher pitch peak (*M* = 421.8 Hz; SD = 1.5 Hz) than the rest of the words, which were presented in a monotone frequency (*M* = 210.7 Hz; SD = 0.6 Hz). The frequency ratio between the peak and the baseline corresponded closely to a musical interval of an octave, with the peak frequency doubling the F0 of the monotonous words. Such large pitch excursions are cross-linguistically typical of IDS (Fernald, 1992).

***Inconsistent pitch peak.*** An octave pitch peak was applied randomly to one word of the utterance, with the condition that each word of the artificial language was stressed at least once and no more than twice. To avoid the *absence* of pitch cue providing a cue this was the target, each target label was also stressed, but only once over the training. Thus, in this condition, pitch emphasis was inconsistent with the co-occurrence cue between the target label and its correspondent image category (Morgan et al., 1987; Shukla et al., 2011). The focused words were again given an average F0 of 421 Hz (SD = 1.4 Hz), while the rest of the words were presented in monotone (*M* = 210.7 Hz; SD = 0.6 Hz). As in condition 2, the frequency ratio between the pitch peak and the baseline corresponds closely to an interval of an octave.

***Duration..*** he duration of the target label was adjusted to twice that of the non target words (target label: *M* = 800 ms; SD = 2 ms; non-target words: *M* = 400 ms; SD = 3 ms). For each word, F0 was normalized to a mean of 210.7 Hz; SD = 0.5 Hz.

***Buzz cue..*** The five-word utterances were those used in condition 1, but now a buzz sound (a low-pass filtered pulse train at 80 Hz, intensity: 67 dB relative to peak) was played during the entire duration of the target label. To prevent clicks, a 20 ms fade-in/fade-out transition was applied the buzz sound. In order to maintain optimal separation of the spoken utterance and the buzz, utterances (including the target label) were played from both stereo channels of the headphones (centering the auditory image), while the buzz sound was played only from the left side.

***Visual cue..*** Again, the same set of utterances as those used in condition 1 were played, but now a visual cue was used during the target label: the “standard” light blue background color surrounding the images was changed to red during the entire duration of the target label.

#### Training and testing procedure

An explicit learning paradigm was used. Participants were randomly assigned to one of the six experimental conditions. They were told that they would participate in an “Alien Language Learning Study” (see Kirby et al., 2008) in which they would see a series of pictures and hear the sounds that an imaginary alien would use to describe those pictures. They were informed that the experiment consisted of a training phase, during which they were simply asked to do their best to understand as much as they could of this language. They were also told that their mastery of the language would be evaluated in a test phase right after the training. After being instructed, participants were seated in a quiet room, at around 60 cm from a 23” monitor (1,920 × 1,080 pixels) and wore Sennheiser HD 520 headphones. The experiment lasted around 8 min.

In both the training and the test phase, the artificial language was manipulated as described above for each condition. To avoid the possibility that some specific image-label correspondences are easier than others, which could bias interpretation, each target label was randomly assigned to an image category across subjects. As illustrated in **Figure 1**, during the training session each utterance was randomly paired with one image (centered on the monitor) including the appropriate referential category, yielding 45 auditory utterance-image pairs (see Yu and Smith, 2007). Each utterance, and each image, was presented only once. The auditory unit-image pairs were presented in a random order across participants. For each slide, playback of the utterance was initiated synchronously with the onset of image presentation. The image remained on screen for a further 1500 ms after the end of the auditory unit’s ∼2s presentation, for a total of approximately 3500 ms per slide.

After the training session, participants received a multiple-choice test. Participants were presented with a novel five-word utterance, containing one of the three target labels, and three novel images simultaneously (one from each category). Each five-word utterance was associated once with a set of three probe images, yielding 45 test trials. The onset of images coincided with the onset of the auditory utterance. The mouse pointer was hidden during sound playback to prevent premature responses. Participants were asked to indicate which image matched the auditory unit by clicking on that image. They could thus make their choice anytime from the end of the auditory stimulus playback up to 4 s after the sound ended. No feedback was provided. An interval of 1 s followed the subject’s response on each trial prior to the onset of the next trial. The order of presentation of the utterance-image trials, as well as the left-to-right arrangement of the three images on the monitor was randomized for each subject. Presenting novel images probes the participants’ ability to apply the acquired reference to members of a potentially infinite set of new images, while the novel utterances examined their ability to process the acquired label within an open-ended set of new utterances.

## RESULTS

Statistical analyses were performed using SPSS for Mac OS X version 19. A binary logistic regression model was built within the generalized linear model framework, to compare overall responses across conditions. Data across all subjects were modeled using a binomial distribution and a logit link function. *Participant ID* was entered as subject variable, *image category* as a within-subject predictor variable and *experimental condition* as a between-group predictor variable. The dependent variable was the proportion of correct choices in participants’ responses (where chance = 33.3%). Five participants were excluded from the analysis because their responses comprised more than 15% timeouts which could not be analyzed. The model provided a good fit (*R*^2^ = 0.65; see Nagelkerke, 1991), and revealed a significant main effect of experimental condition [Wald χ^2^(5) = 28.525, *p* < 0.001], no significant effect of image category [Wald χ^2^(2) = 4.181, *p* = 0.124], and no significant interactions between image category and experimental condition [Wald χ^2^(10) = 11.732, *p* = 0.303]. Consistent with these analyses, a non-parametric Kruskal–Wallis test (with individual responses collapsed across image categories) confirmed that participants’ performance was significantly affected by the experimental condition [*H*(5) = 17.734, *p* = 0.003].

**FIGURE 3:**
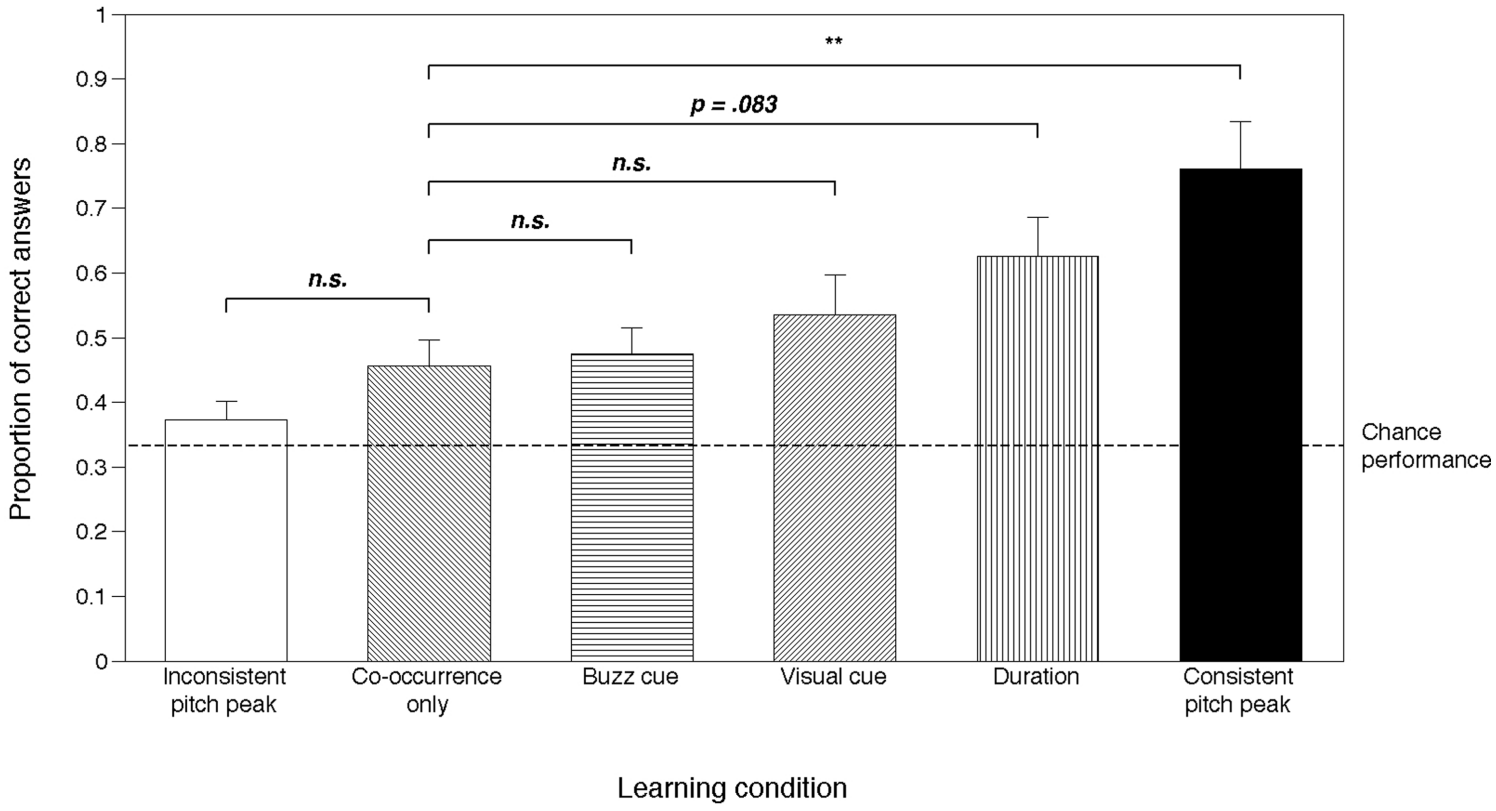
**Percentage of correct responses in each experimental condition.** Error bars represent 95% confidence intervals. Chance performance level is set at 33%. Horizontal lines indicate pairwise comparisons between each condition and the *Co-occurrence only* condition. All conditions except the *Inconsistent pitch peak* were significantly different than chance. *n.s.,* non significant; **p < 0.01.

Pairwise comparisons between the *Co-occurrence only* condition and all the other experimental conditions, using the sequential Bonferroni correction procedure (Holm, 1979), revealed a significant difference only between *Co-occurrence only* and the *Consistent pitch peak* condition [Wald χ^2^(1) = 14.138, *p* = 0.001]. Differences in learning performance did not reach significance between the *Co-occurrence only* condition and the *Duration* condition [Wald χ^2^(1) = 5.351, *p* = 0.083], the *Inconsistent* pitch peak condition [Wald χ^2^(1) = 2.692, *p* = 0.302], the *Visual cue* condition [Wald χ^2^(1) = 1.246, *p* = 0.529], or the *Buzz cue* condition [Wald χ^2^(1) = 0.031, *p* = 0.860]. Thus, only the consistent pitch cueing provided a significant boost in learning efficacy over the ever-present statistical association cue (**Figure 3**).

To further investigate this finding, we calculated the proportionate changes in odds (odds ratio) between the *Co-occurrence only* and all other conditions as a measure of effect size. This analysis revealed that the odds of getting the correct response were 4.851 times higher in the *Consistent pitch peak* condition than in the *Co-occurrence only* condition, while none of the other conditions yielded odds ratios greater than 1.842 times the odds obtained with *Co-occurrence only*, indicating a much stronger effect of the presence of the pitch peak than any of the other attention-highlighting modifications.

A one-sample Wilcoxon signed-rank test was performed for each condition, to test if the median % correct was significantly different from chance (above or below 33.3%). This test revealed that in all experimental conditions except the *Inconsistent pitch peak* condition, percent correct was significantly higher than expected by chance (*Co-occurrence only* condition: *z* = 2.951, *p* = 0.003; *Consistent pitch peak* condition: *z* = 3.473, *p* = 0.001; *Duration* condition: *z* = 3.816, *p* < 0.001; *Visual cue* condition: *z* = 2.951, *p* = 0.003; *Buzz cue* condition: *z* = 3.286, *p* = 0.001). For the *Inconsistent pitch peak* condition, participant performance did not differ significantly from chance (*z* = 1.645, *p* = 0.100).

## DISCUSSION

We found that performance in the EIM task was significantly higher than that observed with simple co-occurrence *only* when pitch prominence consistently marked the target label. Successful learning of target labels occurred in all conditions except the *Inconsistent Pitch Peak* condition. The fact that participants performed above chance in the *Co-occurrence only* condition shows that consistent cross-modal co-occurrence between target labels and their referents was sufficient to allow word learning, consistent with previous research on statistical cross-modal coherence in learning labels for individual objects (Gogate and Bahrick, 1998).

Comparisons between the *Co-occurrence only* condition and the other experimental conditions showed that only one condition yielded a significant increase in performance: the *Consistent pitch peak* condition. When duration, screen color change, or buzz cues were used to highlight attention to the target label, no significant increase in learning performance was observed (although a trend at *p* = 0.083 was seen for *Duration*). These results provide compelling evidence that, of all the cues examined here, *only* exaggerated pitch contour values typical of IDS are salient enough that, if used as markers of the target label, and thus of statistical cross-modal regularities, they significantly aid word learning. Although the manipulation of duration, visual and non-prosodic acoustic cues were quite extreme (especially the visual cue), they did not significantly improve participants’ performance in word acquisition over simple cross-modal statistical regularities, strongly suggesting that the pitch effect demonstrated here goes beyond any general attentional effects (i.e., “von Restorff” effects).

Previous research has shown that the positive effects of IDS typical parameters such as prominent pitch values, exaggerated formant space (vowel hyperarticulation) and/or grammatical simplicity can assist spoken word identification (Sherrod et al., 1977; Burnham et al., 2002; Thiessen et al., 2005). Our results extend these findings, demonstrating a positive didactic effect of IDS-typical pitch prominence in the complex process of word learning as operationally defined here, and contrast with the suggestion that pitch highlighting has no positive didactic effects (Uther et al., 2007). Future work should evaluate the role of vowel hyperarticulation or grammatical simplicity in this task.

Given that the performance in the *Duration* condition was marginally significantly higher than in the *Co-occurrence only* condition, our data is compatible with findings indicating that prominent lengthening of utterances and/or specific words at the ends of utterances can also assist learners in communicative tasks (Church et al., 2005). The trend in our data suggests that, with larger samples or more extensive training, duration might also show a significant augmentation of word learning. However, our odds ratios comparisons suggest a stronger role of pitch prominence relative to timing as a learning booster, mirroring the findings of (Seidl, 2007). Our findings support the hypothesis that the natural predisposition to perceive cross-modal regularities, and the exposure to prosodically highlighted stimuli, are intertwined aspects of language learning (Christiansen and Dale, 2001).

Regarding the *Inconsistent pitch peak* condition, the impairment or lack of learning improvement compared with the *Co-occurrence only* condition demonstrates that the mere presence of pitch exaggerated contours somewhere in an utterance does not aid learning. This finding suggests that pitch enhancement might override cross-situational statistical learning, being a more salient cue for adult participants. Furthermore, evidence on this condition indicates that improved learning is not simply due to a general increased attentiveness induced by the presence of an arousing pitch peak (Fernald, 1992).

It is notable that the addition of a visual cue synchronous with the target label did not significantly improve learning performance in comparison to the *Co-occurrence only* condition. This is consistent with some previous research on the interpretation of pragmatic cues as intentional acts of reference. Neither simply pointing to an intended referent (Grassmann and Tomasello, 2010), or highlighting it with a flashlight and a general attentional phrase (Keates and Graham, 2008), is sufficient for correct label acquisition. In these cases, what makes a communicative act is its intentional connotation, i.e., the interlocutors’ ability to engage in joint attention frames of reference (Tomasello, 2000).

Importantly, our results suggest that adults exploit pitch enhancement as a pragmatic cue to relevant similarities among referents across multiple visual contexts. This finding contributes to a research framework that warrants further work: the effect of prosodic modulation as an invitation to generate referential categories across multiple visual environments in spoken interactions.

Our results may also have implications for models of language evolution, and are compatible with the suggestion that the increased use of prosodic and gestural modifications typical of motherese might have been a useful cue that made vocal language easier to process for hominins (Falk, 2004; de Boer, 2005a,b). Our data suggest possible links between two crucial hypotheses in the literature on the evolution of language: (a) Darwin’s hypothesis that a music-like modulation of voice had a special role in the initial evolution of verbal language (Darwin, 1871) and (b) the hypothesis that mutual segmentation of speech streams and situational contexts initiated a subsequent evolutionary process of linguistic elaboration (Wray, 1998; Okanoya and Merker, 2007).

Some limitations of the current study are worth noting. First, we used adult participants who, unlike neonates, already know that a language-learning task implies the association between sounds and referents. Future work should examine the learning effects of attentional highlighting markers for preverbal infants, who presumably do not possess the mental categories employed here (humans, animals, and mountains). Future work could also examine novel referential categories in adults, and again evaluate the effects of pitch and other cues in guiding the formation of *new* categories.

There are many ways in which the paradigm introduced here can be extended. The simple paradigm used here lacks any syntactic relation between units, a property that no doubt plays an important role in word learning. Future studies might include multiple target labels in each utterance, or investigate the ability to map words to referents of different kinds (e.g., nouns versus verbs, or statements versus requests). This could provide new insights into how prosodic highlighting interacts with the syntactic and semantic organization of the utterance, and *vice versa* (bootstrapping process). Moreover, one could investigate additional statistical information with our design, utilizing, for example, multi-syllabic words defined by transition probabilities between syllables, rather than the monosyllabic target labels we employed. Image complexity could also be manipulated (e.g., referent size, number of distractors, or emotional connotation). Finally, it would be interesting to employ our task in animals, or using wordless melodies (rather than speech). Clearly, the paradigm introduced here opens up multiple research avenues to investigate word-learning across contexts in a controlled, yet naturalistically complex, experimental environment. We hope that further research along these lines will lead to a richer understanding of the complex cognitive processes involved in language acquisition.

## AUTHOR CONTRIBUTIONS

Piera Filippi developed the study concept. All authors contributed to the study design. Piera Filippi performed testing and data collection. Piera Filippi and Bruno Gingras performed data analysis. Piera Filippi drafted the manuscript, and Bruno Gingras and W. Tecumseh Fitch provided critical revisions. All authors approved the final version of the manuscript for submission.

## SUPPLEMENTARY MATERIAL

The Supplementary Material for this article can be found online at: http://www.frontiersin.org/journal/10.3389/fpsyg.2014.01468/abstract">

## Conflict of Interest Statement

The authors declare that the research was conducted in the absence of any commercial or financial relationships that could be construed as a potential conflict of interest.

## References

[B1] Aslin R. N. Woodward J. Z. LaMendola N. P. Bever T. G. (1996). “Models of word segmentation in maternal speech to infants,” in *Signal to Syntax* eds Morgan J. L. Demuth K. (Hillsdale, NJ: Erlbaum), 117–134.

[B2] Baldwin D. (1993). Early referential understanding: infants’ ability to recognize referential acts for what they are. *Dev. Psychol.* 29 832–843 10.1037/0012-1649.29.5.832

[B3] Bloom P. (2000). *How Children Learn the Meanings of Words.* Cambridge, MA: MIT Press.

[B4] Boersma P. Weenink D. (2007). *PRAAT.* Dallas, TX: Summer Institute of Linguistics Available at:

[B5] Brown R. (1958). *Words and Things.* Glencoe, IL: Free Press.

[B6] Burnham D. Kitamura C. Vollmer-Conna U. (2002). What’s new, pussycat? On talking to babies and animals. *Science* 296 1435–1435 10.1126/science.1069587 12029126

[B7] Chomsky N. (2000). *New Horizons in the Study of Language and Mind.* Cambridge: Cambridge University Press 10.1017/CBO9780511811937

[B8] Christiansen M. H. Dale R. A. C. (2001). “Integrating distributional, prosodic and phonological information in a connectionist model of language acquisition,” in *Proceedings of the 23rd Annual Conference of the Cognitive Science Society* (Mahwah, NJ: Lawrence Erlbaum Associates), 220–225.

[B9] Church R. Bernhardt B. Pichora-Fuller K. Shi R. (2005). Infant-directed speech: final syllable lengthening and rate of speech. *Can. Acoust.* 33 13–19.

[B10] Darwin C. (1871). *The Descent of Man, and Selection in Relation to Sex*. London: John Murray 10.1037/12293-000

[B11] de Boer B. (2005a). Evolution of speech and its acquisition. *Adapt. Behav.* 13 281–292 10.1177/105971230501300405

[B12] de Boer B. (2005b). “Infant directed speech and the evolution of language,” in *Evolutionary Prerequisites for Language* ed. Tallerman M. (Oxford: Oxford University Press), 100–121.

[B13] Falk D. (2004). Prelinguistic evolution in early hominins: whence motherese? *Behav. Brain Sci.* 27 491–502 10.1017/S0140525X04000111 15773427

[B14] Fernald A. (1992). “Human maternal vocalizations to infants as biologically relevant signals: an evolutionary perspective,” in *The Adapted Mind: Evolutionary Psychology and the Generation of Culture* eds Barkow J. H. Cosmides L. E. Tooby J. E. (New York NY: Oxford University Press), 389–428.

[B15] Fernald A. Mazzie C. (1991). Prosody and focus in speech to infants and adults. *Dev. Psychol.* 27 209–221 10.1037/0012-1649.27.2.209

[B16] Gleitman L. (1990). The structural sources of verb meanings. *Lang. Acquis.* 1 1–55 10.1207/s15327817la0101_2

[B17] Gogate L. J. Bahrick L. E. (1998). Intersensory redundancy facilitates learning of arbitrary relations between vowel sounds and objects in seven-month-old infants. *J. Exp. Child Psychol.* 69 133–149 10.1006/jecp.1998.2438 9637756

[B18] Gomez R. L. Gerken L. (2000). Infant artificial language learning and language acquisition. *Trends Cogn. Sci.* 4 178–186 10.1016/S1364-6613(00)01467-4 10782103

[B19] Grassmann S. Tomasello M. (2010). Young children follow pointing over words in interpreting acts of reference. *Dev. Sci.* 13 252–263 10.1111/j.1467-7687.2009.00871.x 20121881

[B20] Grieser D. L. Kuhl P. K. (1988). Maternal speech to infants in a tonal language: support for universal prosodic features in motherese. *Dev. Psychol.* 24 14–20 10.1037/0012-1649.24.1.14

[B21] Holm S. (1979). A simple sequentially rejective multiple test procedure. *Scand. J. Stat.* 6 65–70 10.2307/4615733

[B22] Johnson E. K. Jusczyk P. W. (2001). Word segmentation by 8-month-olds: when speech cues count more than statistics. *J. Mem. Lang.* 44 548–567 10.1006/jmla.2000.2755

[B23] Keates J. Graham S. A. (2008). Category markers or attributes: why do labels guide infants’ inductive inferences? *Psychol. Sci.* 19 1287–1293 10.1111/j.1467-9280.2008.02237.x 19121139

[B24] Kirby S. Cornish H. Smith K. (2008). Cumulative cultural evolution in the laboratory: an experimental approach to the origins of structure in human language. *Proc. Natl. Acad. Sci.* *U.S.A.* 105 10681–10686 10.1073/pnas.0707835105 PMC2504810 18667697

[B25] Kuhl P. K. (2004). Early language acquisition: cracking the speech code. *Nat. Rev. Neurosci.* 5 831–843 10.1038/nrn1533 15496861

[B26] Markman E. M. (1990). Constraints children place on word learning. *Cogn.* *Sci.* 14 57–77 10.1207/s15516709cog1401_4

[B27] Medina T. N. Snedeker J. Trueswell J. C. Gleitman L. R. (2011). How words can and cannot be learned by observation. *Proc. Natl. Acad. Sci. U.S.A.* 108 9014–9019 10.1073/pnas.1105040108 21576483 PMC3107260

[B28] Morgan J. L. Meier R. P. Newport E. L. (1987). Structural packaging in the input to language learning: contributions of prosodic and morphological marking of phrases to the acquisition of language. *Cogn. Psychol.* 19 498–550 10.1016/0010-0285(87)90017-X 3677585

[B29] Nagelkerke N. J. D. (1991). A note on a general definition of the coefficient of determination. *Biometrika* 78 691–692 10.1093/biomet/78.3.691

[B30] Nurmsoo E. Bloom P. (2008). Preschoolers’ perspective taking in word learning do they blindly follow eye gaze? *Psychol. Sci.* 19 211–215 10.1111/j.1467-9280.2008.02069.x 18315790

[B31] Okanoya K. Merker B. (2007). “Neural substrates for string-context mutual segmentation: a path to human language,” in *Emergence of Communication and Language* eds Lyon C. Nehaniv C. Cangelosi A. (London: Springer-Verlag), 421–434.

[B32] Quine W. V. O. (1960). *Word and Object* Cambridge, MA: MIT Press.

[B33] Seidl A. (2007). Infants’ use and weighting of prosodic cues in clause segmentation. *J. Mem. Lang.* 57 24–48 10.1016/j.jml.2006.10.004

[B34] Seidl A. Tincoff R. Baker C. Cristia A. (2014). Why the body comes first: effects of experimenter touch on infants’ word finding. *Dev. Sci.* 1–10 10.1111/desc.12182 24734895

[B35] Sherrod K. B. Friedman S. Crawley S. Drake D. Devieux J. (1977). Maternal language to prelinguistic infants: syntactic aspects. *Child Dev.* 48 1662–1665 10.2307/1128531 608376

[B36] Shukla M. White K. S. Aslin R. N. (2011). Prosody guides the rapid mapping of auditory word forms onto visual objects in 6-mo-old infants. *Proc. Natl. Acad. Sci. U.S.A.* 108 6038–6043 10.1073/pnas.1017617108 21444800 PMC3076873

[B37] Thiessen E. D. Hill E. A. Saffran J. R. (2005). Infant-directed speech facilitates word segmentation. *Infancy* 7 53–71 10.1207/s15327078in0701_5 33430544

[B38] Tomasello M. (2000). The social-pragmatic theory of word learning. *Pragmatics* 10 401–413.

[B39] Uther M. Knoll M. A. Burnham D. (2007). Do you speak E-NG-LI-SH? A comparison of foreigner-and infant-directed speech. *Speech Commun.* 49 2–7 10.1016/j.specom.2006.10.003

[B40] Vlach H. A. Sandhofer C. M. (2014). Retrieval dynamics and retention in cross-situational statistical word learning. *Cogn. Sci.* 38 757–774 10.1111/cogs.12092 24117698 PMC3979515

[B41] Wagner M. Watson D. G. (2010). Experimental and theoretical advances in prosody: a review. *Lang. Cogn. Process.* 25 905–945 10.1080/01690961003589492 22096264 PMC3216045

[B42] Waxman S. R. Braun I. (2005). Consistent (but not variable) names as invitations to form object categories: new evidence from 12-month-old infants. *Cognition* 95 B59–B68 10.1016/j.cognition.2004.09.003 15788158

[B43] Waxman S. R. Gelman S. A. (2009). Early word-learning entails reference, not merely associations. *Trends Cogn. Sci.* 13 258–263 10.1016/j.tics.2009.03.006 19447670 PMC2829659

[B44] Wittgenstein L. (2009). *Philosophical Investigations* trans. Anscombe G. E. M. Hacker P. M. S. Schulte J. Oxford: Wiley Blackwell (Original work published 1953).

[B45] Wray A. (1998). Protolanguage as a holistic system for social interaction. *Lang. Commun.* 18 47–67 10.1016/S0271-5309(97)00033-5

[B46] Yu C. Smith L. B. (2007). Rapid word learning under uncertainty via cross-situational statistics. *Psychol. Sci.* 18 414–420 10.1111/j.1467-9280.2007.01915.x 17576281

