# Supplementary material for: Pitch enhancement facilitates word learning across visual contexts
Source: Front Psychol. 2014 Dec 22;5:1468. doi: 10.3389/fpsyg.2014.01468 (PMC4273622; doi:10.3389/fpsyg.2014.01468)
Supplement: Supplementary file 1 [file Table_1.PDF]

**GA**kenasufo	**GA**puladero	**GA**najifoke
fo**GA**jinake	ji**GA**purola	su**GA**fokezi
ropu**GA**jide	dena**GA**fosu	suzi**GA**roke
lakefo**GA**ji	desuna**GA**fo	narozi**GA**de
purolade**GA**	fonakesu**GA**	rolazipu**GA**

**LU**kenasufo	**LU**puladero	**LU**najifoke
fo**LU**jinake	ji**LU**purola	su**LU**fokezi
ropu**LU**jide	dena**LU**fosu	suzi**LU**roke
lakefo**LU**ji	desuna**LU**fo	narozi**LU**de
purolade**LU**	fonakesu**LU**	rolazipu**LU**

**MI**kenasufo	**MI**puladero	**MI**najifoke
fo**MI**jinake	ji**MI**purola	su**MI**fokezi
ropu**MI**jide	dena**MI**fosu	suzi**MI**roke
lakefo**MI**ji	desuna**MI**fo	narozi**MI**de
purolade**MI**	fonakesu**MI**	rolazipu**MI**

Table 1. Artificial language in the following conditions: *Co-occurrence only*, *Consistent pitch peak*, *Duration*, *Visual cue*, *Buzz cue*. Target labels are capitalized and bold.
