# Supplementary material for: Pitch enhancement facilitates word learning across visual contexts
Source: Front Psychol. 2014 Dec 22;5:1468. doi: 10.3389/fpsyg.2014.01468 (PMC4273622; doi:10.3389/fpsyg.2014.01468)
Supplement: Supplementary file 2 [file Table_2.PDF]

**ga**KEnasufo	**ga**puladeRO	**ga**NAjifoke
FO**ga**jinake	JI**ga**purola	su**GA**fokezi
roPU**ga**jide	dena**ga**FOsu	SUzi**ga**roke
lakefo**ga**JI	DEsuna**ga**fo	narozi**ga**DE
puroLAde**ga**	fonakeSU**ga**	rolaZIpu**ga**

**lu**KEnasufo	**lu**puladeRO	**lu**NAjifoke
FO**lu**jinake	JI**lu**purola	su**LU**fokezi
roPU**lu**jide	dena**lu**FOsu	SUzi**lu**roke
lakefo**lu**JI	DEsuna**lu**fo	narozi**lu**DE
puroLAde**lu**	fonakeSU**lu**	rolaZIpu**lu**

**mi**KEnasufo	**mi**puladeRO	**mi**NAjifoke
FO**mi**jinake	JI**mi**purola	su**MI**fokezi
roPU**mi**jide	dena**mi**FOsu	SUzi**mi**roke
lakefo**mi**JI	DEsuna**mi**fo	narozi**mi**DE
puroLAde**mi**	fonakeSU**mi**	rolaZIpu**mi**

Table 2. Artificial language in the *Inconsistent pitch peak* condition. Bold syllables are the target labels, while the capitalized syllables are stressed in pitch.
